# Evaluation of the Effectiveness of Modified Pectoral Nerve Blocks Type II (PECS II) for Vascular Access Port Implantation Using Cephalic Vein Venesection

**DOI:** 10.3390/jcm10245759

**Published:** 2021-12-09

**Authors:** Jarosław Janc, Marek Szamborski, Artur Milnerowicz, Lidia Łysenko, Patrycja Leśnik

**Affiliations:** 1 Department of Anaesthesiology and Intensive Therapy, 4th Military Clinical Hospital, 50-981 Wroclaw, Poland; jjanc@4wsk.pl (J.J.); marek.szamborski@gmail.com (M.S.); lily4470@gmail.com (L.Ł.); 2 Department of Vascular Surgery, 4th Military Clinical Hospital, 50-981 Wroclaw, Poland; milnerowicz.artur@gmail.com

**Keywords:** pectoral nerve block type II, Port-A-Cath implantation, vascular access, port implantation procedure, venesection method, pain intensity, patient satisfaction, operator’s condition

## Abstract

The vascular access port implantation procedure can be performed using the venesection method by inserting a catheter into the cephalic vein in the region of the deltopectoral groove. This method eliminates the need for catheter tunneling. An alternative method to infiltration anaesthesia for port implantation may be a modified pectoral nerve block type II (PECS II). This study aimed to evaluate the effectiveness of modified PECS II for vascular access port implantation using cephalic vein venesection. This retrospective observational study was conducted at the 4th Military Clinical Hospital in Wroclaw, Poland. A group of 114 patients underwent the modified PECS II block and additional cutaneous infiltration anesthesia at the incision line. Pain intensity was assessed on the NRS scale measured intraoperatively at four points. The QoR-15 questionnaire was used to assess patient satisfaction during the first 24 h after surgery. The operator’s condition assessment score was used to assess surgical conditions and operator comfort. The analysis showed that the median pain intensity during vascular port implantation was 0. A statistically significant difference in pain intensity was demonstrated between the specialist’s group and the resident’s group at the second and third measurement points (*p* < 0.008; *p* < 0.012). The mean value on the QoR-15 scale was 132. There was a significant difference between the pain scores of the groups. The mean score in the pain position in the specialist’s group was 18 points and in the resident’s group, it was 19 points (*p* < 0.029). In conclusion, the present study revealed that the modified PECS II block is an effective and safe method of anesthesia for Port-A-Cath implantation.

## 1. Introduction

Vascular access port implantation procedures are mainly performed under local infiltration anesthesia. A common technique is venipuncture of the right or left internal jugular vein with insertion of the catheter into the superior vena cava, at the border of its junction with the right atrium [1,2]. The procedure of vascular access port implantation can also be performed by venesection, inserting the catheter into one of the veins of the arm in the region of the deltopectoral groove. In most cases, it is the cephalic vein. The port chamber is implanted into the subcutaneous pocket created in the ipsilateral subclavian region [3,4]. This method eliminates the need for catheter tunnelling. It is also possible to implant a vascular access port catheter by performing subclavian vein venipuncture [1].

An alternative method of infiltration anesthesia for vascular access port implantation is the use of regional compartmental pectoral nerve block type II (PECS II) block [5]. PECS II is a modification of PECS I block, in which, after local anesthesia (LA) deposition between the pectoral muscles, a dose of local anesthetic is additionally administered to the fascial compartment between the pectoralis minor muscle and the serratus anterior muscle, blocking the lateral branches of the intercostal nerves Th2–4, the intercostobrachial nerve, and the long thoracic nerve [6,7]. PECS II can be used for the procedures described above as well as for more extensive breast surgery procedures (mastectomy, quadrantectomy) [8]. For the identification of fascial spaces, the ultrasound technique is used, with a linear transducer (5–12 MHz).

The aim of this study was to evaluate the effectiveness and safety of the modified PECS II block in anesthesia for vascular access port implantation procedures using cephalic vein venesection. In addition, patient comfort during and after the procedure and operator comfort during the vascular access port implantation procedure were assessed, also in the case of procedures lasting more than 60 min and in obese patients. The primary hypothesis of the study was that the PECS II block would be an effective and safe method of anesthesia regarding risk and complications’ reduction for the vascular access port implantation procedure using the venesection method. The secondary hypothesis was that the PECS II block would provide optimal comfort for the patient and operator even during lengthy port implantation procedures and in obese patients.

## 2. Materials and Methods

### 2.1. Study Design and Participants

The study was designed as a single-center, retrospective, and observational study. All the patients signed written informed consents for the PECS II block procedure. The study included adult patients of both sexes, aged 22 to 82 years with ASA (American Society of Anesthesiologist) II–IV [9], treated at the Clinical Oncology Department of the 4th Military Clinical Hospital in Wroclaw in the period from 1 April 2019 to 30 June 2020, qualified for planned vascular access port implantation for treatment with intravenous cycles of chemotherapy for cancer. All the included patients completed a postoperative Quality of Recovery-15 (QoR-15) questionnaire [10].

The exclusion criteria were as follows: patient under 18 years of age, lack of patient consent, coagulation disorder, known allergy to the studied anesthetics drugs, inflammation at the site of the planned block, missing data in the research protocol, or technical difficulties in the implementation of the block.

**Figure 1:**
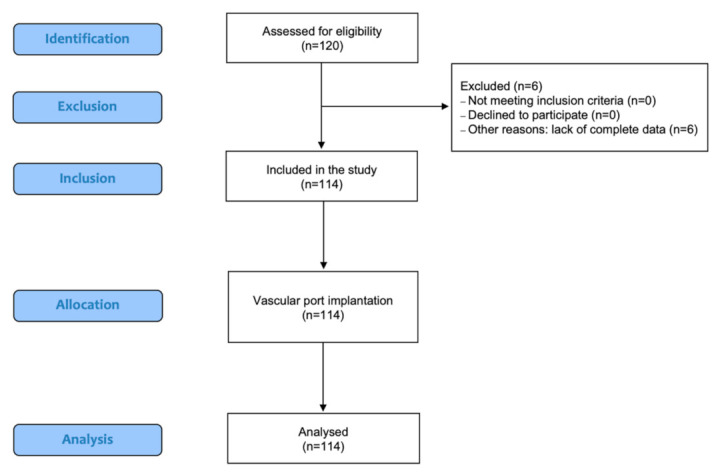
STROBE flow chart of the study participants.

The study was approved by the Bioethical Committee of the Military Medical Chamber in Warsaw, Poland (approval no. KB–1/21). The study was conducted in accordance with the guidelines of the Declaration of Helsinki and Good Clinical Practice. The Strengthening the Reporting of Observational Studies in Epidemiology (STROBE) guidelines were followed and the flow chart is shown in Figure 1.

### 2.2. Application of PECS II and Port Catheter

For all the patients included in the study, the implantation of the vascular access port was planned through cephalic venesection in the area of the deltopectoral groove on the right or left side of the chest. The right side was selected by default to shorten the length of the vascular catheter. The vascular access port was implanted on the left side in the case of contraindications to implantation on the right side. In the event of anatomical obstacles or technical problems during the venesection, modification of the procedure was performed in the form of subclavian venipuncture. No pharmacological premedication before or analgosedation during the procedure were planned. The treatments were performed by a steady (consistent) team of doctors: an anesthesiology and intensive care specialist with 28 years of work experience and extensive experience in the field of vascular access port implantation, and, under the direct supervision of the above-mentioned specialist, a resident doctor in the fourth year of training in anesthesiology and intensive care, with basic skills in the field of vascular access port implantation and three years of experience in regional blocks.

The implantation procedure was performed in the modified PECS II block. The modification consisted in USG-guided injection of an anesthetic between the pectoralis major and pectoralis minor muscle fascia and into the interfascial space under the pectoralis minor muscle assuming that the deposited anesthetic, through the interfascial plane, would reach the structures located more laterally (into the fascia of the serratus anterior muscle), blocking the lateral branches of intercostal nerves Th2–4, the intercostobrachial nerve, and the long thoracic nerve. Compared to the classic PECS II method, these structures were identified more medially, i.e., in the region of the mid-clavicular line. The intended volume of anesthetic (from 15–20 mL) was deposited evenly between both compartments.

**Figure 2:**
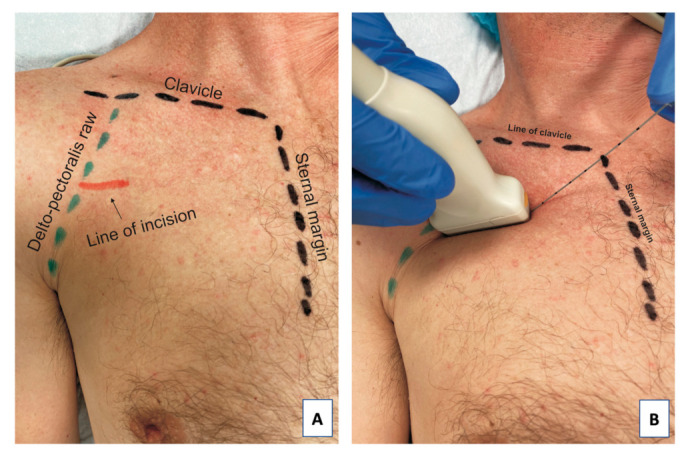
Anatomical structures before the procedure: the red line marks the line of skin incision (**A**) and the position of the ultrasound transducer and direction of the needle during the PECS II block (**B**).

The vascular access port implantation procedure began with aseptic preparation of the surgical field. Just below the clavicle, muscle groups and fascial structures were identified with the ultrasound transducer (Sonoscanner U-Lite with the 5–12 Mhz linear transducer, Portable version). After identifying the structures under ultrasound guidance, with the in-plane technique, a guiding needle (Echoplex <Vygon> with a diameter of 22 Ga and length of 50 mm) was inserted parallel to the clavicle towards the head of the humerus at an angle of 30–45° (Figure 2) at the level of the third rib.

**Figure 3:**
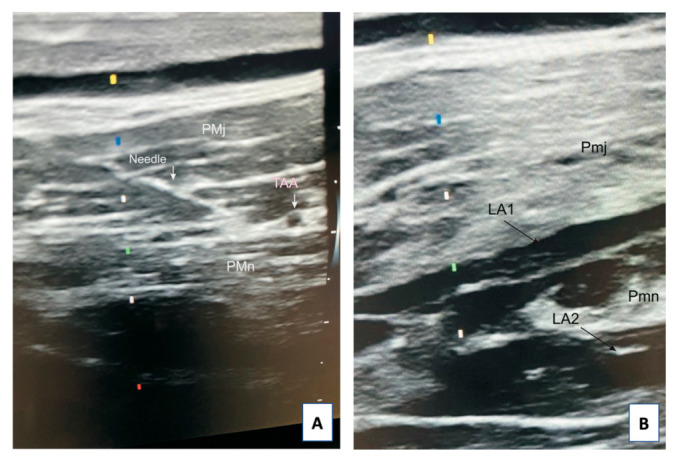
Sonogram of the needle position during the application of the modified PECS II (**A**) and local anesthetic deposition after application of the modified PECS II block (**B**). Abbreviations: Pmj, pectoralis major muscle, Pmn, pectoralis minor muscle, TAA, thoracoacromial artery, LA1, local anesthetic spread into the fascial plane between Pmj and Pmn, LA2, local anesthetic spread into the fascial plane underneath Pmn.

After confirming that the intended space was reached, 8–10 mL of the drug were deposited in a volume sufficient to delaminate the fascial lamellae over a length of at least 4 cm, thus anaesthetizing the neurovascular structures located there. Subsequently, deeper structures (under the pectoralis minor muscle) were anaesthetized using the same volume of analgesic (Figure 3).

In the technique of vein venesection in the deltopectoral groove, anesthesia of deeper anatomical structures is necessary; however, the lateral thoracic surface does not need to be anaesthetized, which led to the development of the modification of the PECS type II method, adjusting the place of anesthetic deposition to the surgical site.

Due to insufficient skin anesthesia, before the incision, infiltration anesthesia of the incision line was applied using 0.5% lignocaine with 0.005% adrenaline in the volume of 3 mL. Subsequently, in the deltopectoral groove, cephalic vein venesection was performed, and a vascular catheter was introduced into the vein, with its tip placed under fluoroscopy in the superior vena cava at the border with the right atrium. Then, the catheter was connected to the port chamber, which was implanted into the created subcutaneous pocket on the ipsilateral side on the fascia of the pectoralis major muscle. The patient remained under the care of the vascular access port implantation team for 2 h after the procedure. The patient was then discharged home with the recommendation to take oral 500 mg paracetamol (maximum every 6 h/day) if pain occurred within the first 48 h. They were also advised to contact the vascular access port implantation center if pain persisted beyond 48 h.

### 2.3. Analgesia Protocol

The primary outcome measure of the study was the pain intensity during the vascular access port implantation procedure. Since the application of PECS II, each patient was asked about the intensity of pain assessed according to the 11-degree Numerical Rating Scale (NRS) [11,12] at the following measurement points: skin incision, vein preparation, creation of the port chamber pocket, and dressing application. It was assumed that, according to the NRS, low-intensity pain falls within the range of 1–3 points, thus a threshold of ≤3 points was defined as an acceptable NRS level not requiring administration of an additional dose of LA. Pain intensity of ≤1 point at the measurement moments defined the patients as pain-free. The patients also received the NRS questionnaire with the recommendation to record pain intensity for the first 48 h after hospital discharge, once daily and each time before taking paracetamol.

### 2.4. Outcomes Measures

After the procedure, the operator’s comfort during the procedure was assessed using a 3-point questionnaire (operator’s condition assessment). This questionnaire takes into account the following domains: duration of the procedure, cooperation with the patient/stability of the surgical field, presence of complaints of pain during the procedure, and the related need to add LA during the procedure. Responses were rated on a scale of 0–2, with 0 being considered a good rating by the operator, 1 being considered acceptable, and 2 being considered difficult to accept by the operator. The study protocol also recorded the application time of anesthesia, the volume of LA used for infiltration anesthesia and for regional block, the need for intraoperative additional dose of LA, the duration of the procedure, the surgical technique, and perioperative complications until hospital discharge.

At discharge, the patients received the QoR-15 patient satisfaction questionnaire [13] with a recommendation to complete it after 24 h post-discharge. In addition, on postoperative day 7–10, during hospital follow-up connected with suture removal, the patients reported the appearance of any adverse events that occurred up to day 7 after hospital discharge.

### 2.5. Sample Size

STATISTICA version 13.3 (TIBCO Software Inc.) was used for sample size estimations. The minimum sample size in the compared groups (N_min_) was estimated by assuming a minimum significance level of α = 0.05, a test power of 1 − β = 0.8, and the mean values and standard deviations obtained in the pilot study. For these assumptions, the minimum number of patients in each group should be N_min_ = 46. For sample sizes N1 = 64 and N2 = 51, the power of the tests of the parameters that differed significantly was estimated at the level of *p* < 0.05. For the test comparing the level of pain, the power of the test is 1 − β = 0.873. The power of the test comparing the proportion “of venipuncture” is 1 − β = 0.803, and for the significance of the correlation coefficient r between “regional block value” and “BMI”, it is 0.789.

### 2.6. Statistical Analysis

STATISTICA version 13.3 (TIBCO Software Inc., Tulsa, OK, USA) was used for statistical calculations. Continuous quantitative variables were presented as mean values and standard deviations (M ± SD) or, if their distribution deviated from normal, as medians and quartile ranges (Me [Q1; Q3]), while qualitative variables were presented as counts (*n*) and percentages (%). The levels of quantitative variables in the two independent groups were compared using the Student’s *t*-test or Mann–Whitney U test (for nonparametric variables), while qualitative (nominal) variables were compared using the chi-square test. Relationships between two variables were analyzed by calculating Pearson correlation coefficients. The values obtained for the assumed significance level *p* ≤ 0.05 were considered statistically significant.

## 3. Results

Initially, 120 patients who qualified for venous access port implantation for cancer treatment were included in the study. All the patients gave informed consent to participate in the study. Finally, the analysis was conducted in a group of 114 patients. Basic anthropometric data, data on anesthesia, surgery, complications, and paracetamol use after hospital discharge are shown in Table 1.

A statistically significant difference in pain intensity was demonstrated between the specialist’s group and the resident’s group at the second and third measurement points (*p* < 0.05), which is shown in Table 2.

The postoperative QoR-15 patient satisfaction score is shown in Table 3. The mean value on the QoR-15 scale was 132. There was a significant difference between the pain score of the groups. The mean score in the Pain position in the specialist’s group was 18 points and in the resident’s group, it was 19 points.

The conditions and comfort of the operator during the procedure were also assessed. The scale was created by the operators themselves (Table 4).

**Figure 4:**
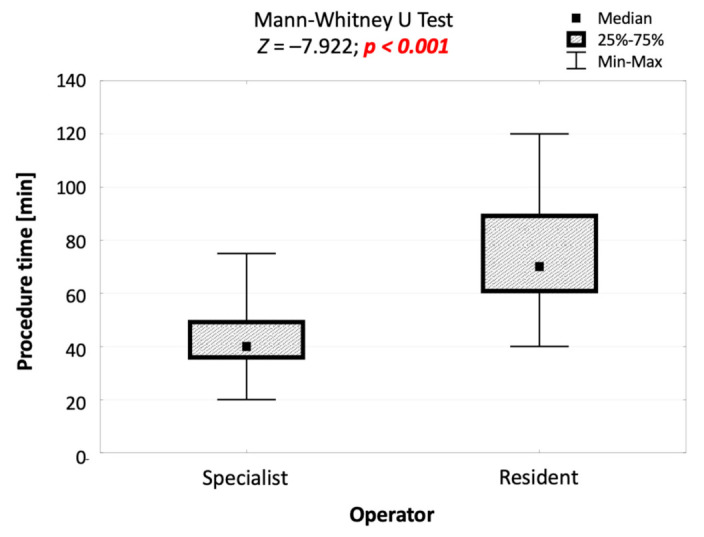
Comparison of the duration of procedures performed by the specialist and by the resident, and the result of the significance test.

The duration of the procedure performed by the resident was statistically significantly longer than the duration of the procedure performed by the specialist by an average of 30 min (70 vs. 40 min; *p* < 0.001; Figure 4).

**Figure 5:**
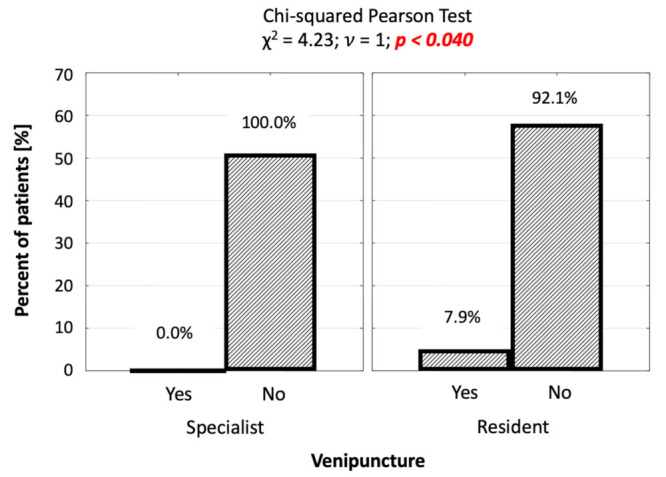
Number (percentage) of patients in groups differing in the performance of venipuncture and the operator, and the result of the independence test.

The percentage of venipuncture in the group of patients operated on by the resident was significantly higher (7.9% vs. 0.0%; *p* = 0.040; Figure 5). Venipuncture was performed in five patients (4.4%), exclusively in the group of procedures performed by the resident.

The correlations of BMI with the amount of LA used to perform the regional block were also analyzed. A significant positive correlation was observed between the patient’s body mass index (BMI) and the volume of LA used for the regional block. The correlation coefficient was r = 0.231 and was significantly different from zero (*p* < 0.014). An increase in BMI of 1 kg/m^2^ was accompanied by an increase in LA volume of 0.14 mL on average.

## 4. Discussion

Implantation of the vascular access port by internal jugular vein venipuncture is simple but requires extensive infiltration anesthesia of soft tissues and tunnelling of the catheter above the clavicle just under the skin from the neck area to the ipsilateral subclavian area. This exposes the catheter to damage, especially at the height of the clavicle and adversely affects the aesthetics of the supraclavicular area. A complication of the above procedure may be post-puncture pneumothorax requiring the insertion of the pleural drain (risk 0.5–6%) or hematoma of the neck (risk 0.5–6.1%), which result in a prolonged stay of the patient in hospital. Puncture of the internal jugular vein also puts the patient at risk of thrombosis or narrowing of the vessel (stenosis) at the site of damage to its wall. Available data in the literature put the risk of such complications at 10% [14,15,16,17].

The procedure of venesection for vascular access port implantation is technically more difficult and requires surgical exposure of the cephalic vein in the deltopectoral groove region. The advantage of this method is a more circumferential insertion of the catheter tip into the venous system, which reduces the risk of damaging the wall of the main veins draining blood from the head and upper limbs, and eliminates the risk of pneumothorax. The disadvantages of this method are the complexity and longer duration of the procedure compared to venipuncture.

Implantation of the catheter by venipuncture of the subclavian vein increases the risk of pneumothorax and damage to the catheter by the guillotine mechanism arising between the clavicle and the first rib (pinch off syndrome). There is also a higher risk of thrombosis and subclavian vein stenosis [16,17]. The advantage of implantation of the vascular access port catheter by cephalic vein venesection and subclavian vein venipuncture is the absence of tunnelling and a significant shortening of the length of the implanted catheter.

The standard method of anesthesia for vascular access port implantation is infiltration anesthesia of the skin and subcutaneous tissue, usually using LA with adrenaline. Intraoperative analgosedation may also be needed with this method of anesthesia [18,19].

The PECS compartmental blocks were introduced into clinical practice by Rafael Blanco [20,21], mainly for perioperative analgesia in breast surgery, but over time, the use of these methods has been extended to various thoracic procedures. Mavarez et al. [22] described the use of PECS I and PECS II compartmental blocks for implantation of electronic cardiac devices (pacemakers, implantable cardioverter-defibrillators (ICDs), cardiac resynchronisation therapy (CRT) pacemakers, and others) with good results [23]. Electrodes were implanted transvenously by venipuncture of the subclavian vein or venesection of the left cephalic vein in the region of the deltopectoral groove [22]. Ince et al. and Munshey et al. [24] also described the successful use of PECS I and PECS II blocks for vascular access port implantation and vascular catheter implantation in children and adults [25].

The use of the PECS II compartmental block, which effectively anaesthetizes deeper anatomical structures, was intended to improve patient comfort by reducing pain sensations during surgical preparation of the cephalic vein. The modification of this method used in this study, whereby the block was performed in the midclavicular line instead of the anterior or mid axillary line, resulted in good-quality intraoperative analgesia. The described PECS II modification is novel approach and was not issued in the available literature.

In our study, the patient pain score on the NRS scale during the procedure at the defined measurement points did not exceed 5 points at measurement point 3, i.e., during the creation of the port pocket. Only in 3 cases was the pain at measuring point 3 equal to 5 and this referred to the patients in the resident’s group. At the other measurement points, the NRS scale value did not exceed 2 points.

A total of 43 cases (37%) were observed to have poorer operator comfort when assessing the procedure duration of over 60 min. The procedure duration was assessed as unacceptable in 1 (1.95%) case in the specialist’s group and in 18 (28.5%) cases in the resident’s group. Although in 24 (38%) cases in the resident’s group the procedure duration was assessed as difficult to accept, no such assessment was made in the specialist’s group. It should be stressed, however, that prolongation of the procedure duration did not significantly reduce the comfort of the patient assessed, which proves that the method of anesthesia was accurately chosen.

In 13 cases (11.4% of patients), worse cooperation with the patient and increased instability of the surgical field related to the patient’s movement were observed. In 11 cases (9.6% of patients), it was determined that an additional dose of LA had to be administered intraoperatively due to the patient experiencing pain. There was no need to add more LA than 3 mL.

Of the complications reported, minor intraoperative bleeding was observed in five patients (4.4%). No allergic reactions were recorded. None of the patients required analgosedation or intravenous analgesics during the procedure. During the check-up of implanted ports and removal of sutures, none of the patients reported any pain discomfort during the 24 h postoperatively; therefore, there was no need to administer paracetamol. There were also no complications or side effects up to 7 days after the procedure.

It needs to be emphasized that performing a PECS II type fascial block alone is not difficult. In the case of our observation, both operators were experienced in performing this type of block using ultrasound; both used similar volumes of LA at similar times. This demonstrates that an operator trained in performing the block, even with shorter professional experience, does not significantly extend the duration the procedure.

For patients with a high BMI, performing effective infiltration anesthesia is more difficult and requires the use of larger volumes of LA. In the analysis presented here, when the modified PECS II fascial block was used, the volume of anesthetic was minimally larger in the group of patients with high BMI. It should be noted that the quality of anesthesia as assessed by the patients on the NRS scale was good. Pai et al. [26] described the use of the PECS-type block for ICD implantation in two very obese patients. It seems to be an effective and safe method of analgesia in the group of patients with high BMI, taking into account a possible increase in LA dose to achieve good intraoperative analgesia.

## 5. Conclusions

The applied modification of the PECS II method, consisting in the identification of structures and deposition of LA in the mid-clavicular line, might be an alternative method of anesthesia for vascular access port implantation using cephalic vein venesection and is equally effective in patients with normal BMI as well as high BMI. This method gave good and safe perioperative analgesia, well-rated both by the patients on the QoR-15 scale and by the operators using the novel operator’s condition assessment score. In addition, the duration of the procedure did not reduce the quality of perioperative analgesia as assessed by the patient intraoperatively on the NRS scale, which may prove the usefulness of this method of anesthesia in clinical practice, particularly for prolonged port implantations performed by learning and less trained physicians.

## Figures and Tables

**Table 1:** Basic anthropometric data, data on anesthesia, surgery, complications, and paracetamol. use after hospital discharge.

Variable	Data
Group size	*N* = 114
Women, *n* (%)	58 (50.9)
Men, *n* (%)	56 (49.1)
Age (years):	
M ± SD	61.0 ± 12.5
Me [Q1; Q3]	63 [53; 70]
Min–Max	22–82
Height (cm)	
M ± SD	167.5 ± 8.8
Me [Q1; Q3]	167 [162; 174]
Min–Max	148–194
Weight (kg)	
M ± SD	70.8 ± 15.2
Me [Q1; Q3]	71 [60; 79]
Min–Max	39–128
BMI (kg/m^2^)	
M ± SD	25.2 ± 4.8
Me [Q1; Q3]	24.3 [22.0; 28.4]
Min–Max	15.5–43.7
ASA score (class) *n (%)*	
2	11 (9.6)
3	93 (81.6)
4	10 (8.8)
Duration of the procedure (min.)	
M ± SD	60.3 ± 23.2
Me [Q1; Q3]	58 [40; 75]
Min–Max	20–120
Volume of LA–regional PECS II block (mL)	
M ± SD	15.2 ± 2.9
Me [Q1; Q3]	15 [15; 18]
Min–Max	10–25
Patients requiring an additional dose of LA *n (%)*	11 (9.6)
Technique of the procedure:	
Venipuncture *n* (%)	5 (4.4)
Venesection *n* (%)	109 (95.6)
Perioperative complications (until hospital discharge) *n (%)*	5 (4.4)
Complications up to 7 days post-hospital discharge *n*	0
Patients requiring paracetamol after hospital discharge *n*	0

Abbreviations: M, mean; SD, standard deviation; Me, median; Q1, lower quartile; Q3, upper quartile; Min, minimum value; Max, maximum value; *n*, number; %, percentage; ASA score; American Society of Anesthesiologists Physical Status Classification System; LA, local anesthetic; PECS II, Pectoral nerve block type II.

**Table 2:** Patient pain assessment on the NRS scale during the procedure at defined measurement points.

NRS	Total (*n* = 114)	Specialist (*n* = 63)	Resident (*n* = 51)	*p* Values
	Me (Min–Max)			
Skin incision/Anesthesia	0.0 (0–2)	0.0 (0–2)	0.0 (0–2)	0.475
Preparation of deltopectoral groove	0.0 (0–2)	0.0 (0–1)	0.0 (0–2)	0.008
Preparation of the port pocket	0.0 (0–5)	0.0 (0–2)	0.0 (0–5)	0.012
Dressing application	0.0 (0–2)	0.0 (0–1)	0.0 (0–2)	0.995

Abbreviations: Me, median; Min, minimum value; Max, maximum value; *n*, number; NRS, Numerical Rating Scale.

**Table 3:** Patient satisfaction during the first 24 h after surgery on the QoR-15 scale including subsequent domains.

QoR-15	Total (*n* = 115)	Specialist (*n* = 63)	Resident (*n* = 51)	*p* Values
	Me (Min–Max)			
Physical comfort	46 (34–50)	46 (38–50)	47 (34–50)	0.723
Emotional status	29 (19–30)	30 (19–30)	29 (19–30)	0.126
Physical independence	20 (20–20)	20 (20–20)	20 (20–20)	-
Psychological support	20 (18–20)	20 (18–20)	20 (20–20)	0.381
Pain	19 (11–20)	18 (11–20)	19 (12–20)	0.029
Total	132 (117–140)	132 (118–140)	133 (117–140)	0.600

Abbreviations: Me, median; Min, minimum value; Max, maximum value; *n*, number; QoR-15, Quality of Recovery-15.

**Table 4:** Surgical conditions and operator comfort on the operator’s condition assessment score and comparison analysis between the specialist and resident.

Operator Scale	Specialist and Resident	Specialist	Resident	*p* Values
	*n* = 114 (100%)	*n* = 51 (44.7%)	*n* = 63 (55.3%)	
Duration of the procedure				
0 (procedure duration ≤ 60 min.)	71	50	21	
1 (procedure duration > 60n ≤ 80 min).	19	1	18	<0.001
2 (procedure duration > 80 min.)	24	0	24	
Cooperation with the patient/stability of the surgical field				
0 (patient is calm, cooperative)	101	48	53	
1 (patient is restless, moving slightly)	11	3	8	0.193
2 (patient is impatient, moving)	2	0	2	
Pain management/need for adding LA intraoperatively				
0 (no addition of LA)	103	48	55	
1 (adding 3 mL of LA)	11	3	8	0.365
2 (adding > 3 mL of LA)	0	0	0	

Abbreviations: *n*, number; LA, local anesthesia. Assessment scale: 0—good; 1—acceptable; 2—difficult to accept.

## Data Availability

The data presented in this study are available on request from the corresponding author (P.L.).
